# Information, communication, and cancer patients’ trust in the physician: what challenges do we have to face in an era of precision cancer medicine?

**DOI:** 10.1007/s00520-020-05692-7

**Published:** 2020-09-03

**Authors:** Theresia Pichler, Amy Rohrmoser, Anne Letsch, C. Benedikt Westphalen, Ulrich Keilholz, Volker Heinemann, Mario Lamping, Philipp J. Jost, Kristina Riedmann, Peter Herschbach, Ute Goerling

**Affiliations:** 1 Comprehensive Cancer Center Munich, partner site TUM, Klinikum rechts der Isar, Munich, Germany; 2 Comprehensive Cancer Center Munich, partner site LMU, University hospital, LMU Munich, Munich, Germany; 3 Charité – Universitätsmedizin Berlin, Charité Comprehensive Cancer Center, Berlin, Germany; 4 Department of Hematology and Oncology, Charité – Universitätsmedizin Berlin, Campus Benjamin Franklin, Berlin, Germany; 5 Department of Medicine III, University Hospital, LMU Munich, Munich, Germany; 6 Center for Personalized Oncology (ZPO), Comprehensive Cancer Center Munich, partner site TUM, Munich, Germany; 7 Medical Department III for Hematology and Oncology, Klinikum rechts der Isar, TUM, Munich, Germany; 8 Munich, Germany

**Keywords:** Oncology, Whole genome sequencing, Precision cancer medicine, Information, Communication, Molecular diagnostic

## Abstract

**Purpose:**

Despite promising achievements in precision cancer medicine (PCM), participating patients are still faced with manifold uncertainties, especially regarding a potential treatment benefit of molecular diagnostics (MD). Hence, MD poses considerable challenges for patient information and communication. To meet these challenges, healthcare professionals need to gain deeper insight into patients’ subjective experiences. Therefore, this qualitative study examined information aspects of MD programs in cancer patients.

**Methods:**

In two German Comprehensive Cancer Centers, 30 cancer patients undergoing MD participated in semi-structured interviews on information transfer and information needs regarding MD. Additionally, patients provided sociodemographic and medical data and indicated their subjective level of information (visual analogue scale, VAS, 0–10).

**Results:**

On average patients had high levels of information (mean = 7, median = 8); nevertheless 20% (*n* = 6) showed an information level below 5 points. Qualitative analysis revealed that patients show limited understanding of the complex background of MD and have uncertainties regarding their personal benefit. Further, patients described unmet information needs. Existential threat in awaiting the results was experienced as burdensome. To withstand the strains of their situation, patients emphasized the importance of trusting their physician.

**Conclusion:**

The challenges in PCM consist in providing unambiguous information, especially concerning treatment benefit, and providing guidance and support. Therefore, psycho-oncology needs to develop guidelines for adequate patient communication in order to help healthcare providers and cancer patients to handle these challenges in the developing field of PCM.

**Electronic supplementary material:**

The online version of this article (10.1007/s00520-020-05692-7) contains supplementary material, which is available to authorized users.

## Background

Rapid progress in the development of novel molecular diagnostic (MD) and therapeutic modalities holds great promise to improve cancer care [1, 2]. However, as the field of precision cancer medicine (PCM) is still developing, the interweaving of translational research and clinical practice [3] poses considerable challenges for patient information transfer and communication. With the broad use of comprehensive genomic profiling, PCM has changed the way evidence is generated and how fast new findings are brought into clinical practice [3]. While oncology has historically relied on randomized controlled trials to generate evidence, the complexity of genomics-driven medicine however is only partly addressable in classical clinical trials (the *n* = 1, dilemma, [3]). So far, molecularly guided treatment based on comprehensive molecular profiling across all cancers is largely still exploratory and often lacks satisfying evidence [4, 5]. Even if a genomic alteration is found, only a small percentage of patients gain a substantial treatment benefit from molecularly guided treatment [6–8].

These uncertainties resulting from a lack of evidence are aggravated by missing infrastructure and the clinical characteristics of advanced cancer patients. For patients suffering from advanced stage cancer, maintaining hope in such a vulnerable period of disease seems crucial [9]. As a result, patients show high expectations of a personal treatment benefit [10–12].

Given the manifold uncertainties these patients encounter, questions arise about what information patients need undergoing PCM and how best to communicate it. So far, only a few studies have examined psychosocial implications for patients of PCM programs, and only some of those investigated relevant aspects of information transfer and patient communication in this regard. Many patients have limited understanding of the complex background and/or misinterpret the implications of genomic profiling [13–16]. Over one-third of patients require additional counseling [14]. The review of Wolyniec et al. [17]—analyzing quantitative and qualitative studies on knowledge and information regarding genomic testing—found that in some studies, less than half of patients were able to reflect the procedure and contents of molecular genetic testing. Considering their high expectations, especially regarding a potential treatment benefit [12–14, 18], patients might have difficulties dealing with disappointments. Recent literature emphasizes the need for adequate communication, education, and counseling to ensure informed consent in an era of PCM [10, 11, 19, 20]. So far, an in-depth comprehension of patients’ perspectives on information about PCM in a representative sample prior to disclosure of results of comprehensive molecular profiling that also shed light on potential distress is still pending. Therefore, the aim of this study was to illustrate patients’ subjective needs when undergoing comprehensive genomic profiling and — in this regard — to illuminate potential challenges of communication and information transfer for healthcare providers.

## Methods and materials

### Participants and setting

A qualitative interview study concerning patients’ perspectives on molecular diagnostic was conducted at the Comprehensive Cancer Center Munich (CCC Munich) and the Charité Comprehensive Cancer Center in Berlin (CCCC). Eligible were adult, German-speaking cancer patients who consented to undergo extensive molecular diagnostics of tumor material. We recruited patients from PCM programs which were either whole genome sequencing within a research study or panel sequencing including scientific registry but primarily subjected to clinical practice. For all programs, patients gave informed consent after receiving standardized written and verbal information by a physician.

For both programs, inclusion criteria comprised (a) prevalence of a reliable tumor diagnosis, (b) patients’ refractory to standard therapy, (c) good general condition (ECOG ≤ 2), (d) advanced tumor disease or rare cancer, and (e) recommendation by organ-specific tumor board or treating oncologist. For whole genome sequencing, the following additional criteria had to be fulfilled: younger than or equal to 50 years of age and a life expectancy of more than 6 months. Additionally, patients included in whole genome sequencing programs could choose whether or not they wanted to be informed about identification of possible cancer predisposition syndromes, if there were indications found in the germline. In case of a significant mutation, patients of both programs were preferentially treated as part of a given clinical trial or — where appropriate — would be treated off label. A standardized informed consent and patient information was provided for patients of both programs. According to experts’ ratings, programs were highly comparable. For a detailed description of study methods, see also Rohrmoser et al. [12].

### Study procedure and measures

Data collection took place from November 2017 to December 2018. The interview was accomplished after patients had given informed consent to MD and prior to receiving the results of the analysis. After verbal and written informed consent for the interview study, patients were asked to provide basic sociodemographic and clinical data. Participants were screened for distress using a self-assessment questionnaire. This data has been reported earlier [12] and is not included in the present qualitative evaluation. Furthermore, study assistants explored aspects of information and expectations by means of a semi-structured interview guide (supplementary file 1). In addition, patients indicated on a visual analogue scale how well-informed they felt (0 = “no information,” 10 = “very good information”). The face-to-face interviews lasted approximately 20 min. Interviews were audio-taped and transcribed literally, and personal information was anonymized. The study was approved by the Ethics Committee of the Technical University of Munich (533/17 S), the Ethics Committee of the University of Munich (17-873), and the Ethics Committee of the Charité – Universitätsmedizin Berlin (EA1/137/17).

### Data analyses

Transcripts of interviews were organized using MAXQDA, a software for qualitative analysis [21]. Referring to Kuckartz’ thematic content analysis [22], we used a deductive-inductive approach. We identified relevant passages pertaining to patients’ information regarding MD and its relevance in the current situation of waiting for the results of the MD analysis and grouped it into categories. In doing so, predetermined categories from the interview as well as codes empirically emerging from the data were generated. Code systems were critically reflected and continuously redefined in expert groups within the psychological, medical, and methodological field. IBM SPSS Statistics software package version 25 [23] was used for descriptive analysis.

## Results

### Sample characteristics

Of 33 approached patients, 30 gave informed consent (14 female). Mean age was 46 years (SD = 11.2 years, range = 26–77 years). Most patients had metastases (83%, *n* = 25); illness duration was 28.3 (SD = 45.5) months on average (range = 0–160 months; median = 9 months; for sample details, see Table 1).

**Table 1:** Sociodemographic and medical characteristics of the study patients (*N* = 30) [12]

Patients *n* = 30	Location 1	Location 2	Total (%)
Age			
≤ 50 years	12	10	22 (73)
> 50 years	3	5	8 (27)
Gender			
Male	8	8	16 (53)
Female	7	7	14 (47)
Living status			
Living alone	1	2	3 (10)
Living with partner	13	13	26 (87)
Living with relatives	1	0	1 (3)
Education			
Elementary school	2	2	4 (13)
Junior high	3	7	10 (33)
High school	3	0	3 (10)
Graduated	7	6	13 (43)
Entity of cancer diagnosis			
Gastrointestinal tumor	3	5	8 (27)
Urological tumor	1	4	5 (17)
Neuroendocrine tumor	4	0	4 (13)
Head and neck tumor	3	0	3 (10)
Breast tumor	1	1	2 (7)
Melanoma	0	2	2 (7)
Sarcoma	2	0	2 (7)
Gynecological tumor	0	1	1 (3)
Lung tumor	0	1	1 (3)
Lymphoma	1	0	1 (3)
Brain tumor	0	1	1 (3)
Metastases			
Yes	13	12	25 (83)
No	2	3	5 (17)
Illness duration			
Up to 3 months	3	4	7 (23)
> 3 months to 1 year	5	7	12 (40)
> 1 year to 5 year	4	2	6 (20)
> 5 years	3	2	5 (17)
Prior treatments			
Surgery	8	6	14 (47)
Radiotherapy	6	7	13 (43)
Chemotherapy	10	12	22 (73)
Immunotherapy	3	3	6 (20)

### Special aspects of information management regarding molecular diagnostics

**Fig. 1:**
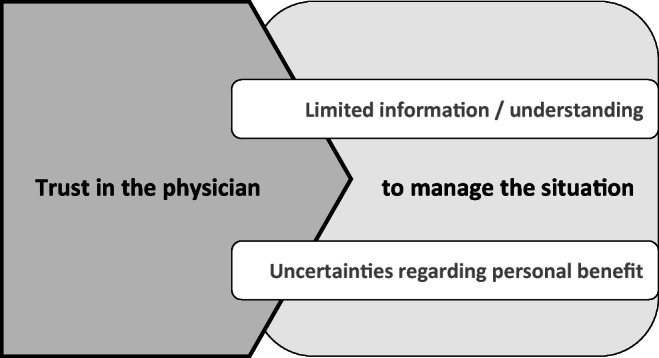
Information and communication regarding the novel molecular research approach

Three aspects were identified as particularly characteristic for information and communication regarding the novel molecular research approach (Fig. 1): (a) limited understanding of and limited information about the complex background of PCM, (b) uncertainties regarding personal benefits of PCM, and (c) the central role that the physician played to manage patients’ challenging situation while awaiting the results.

#### Limited understanding and information of the complex background of PCM

Results of the visual analogue scale on patients’ subjective level of information revealed that on average, patients felt to have a high level of information (mean = 7, median = 8, SD = 2, range = 1.5–10); nonetheless 20% (*n* = 6) indicated an information level below 5 points. These highly heterogeneous subjective information levels were also found in the qualitative data. Patients indicated that they had a lay understanding of PCM in a way that they understood the basic approach but found the details and background of tumor genomic profiling to be too complex for them to understand. However, patients indicated that these details were not relevant to them.

(…) it was understandable to me. Probably not down to the last detail, but I know now what it's about and how it is analyzed, and so I think it’s enough for me. (IP9)

While most patients knew about the organizational process relevant to them, some missed information about the next steps after providing their tumor tissue, if and in what way they would be contacted after results were available and the actual kind of treatment resulting therefrom:

What happens after that? Will I be contacted? Will, uh, will any medication be sent to me? Uh, mh, how… how, does that work? Do I have to come back here? Is it a... infusion? Is it chemo? Is it...? Well, this…this section is somehow still... not so clear to me. (IP12)

**Fig. 2:**
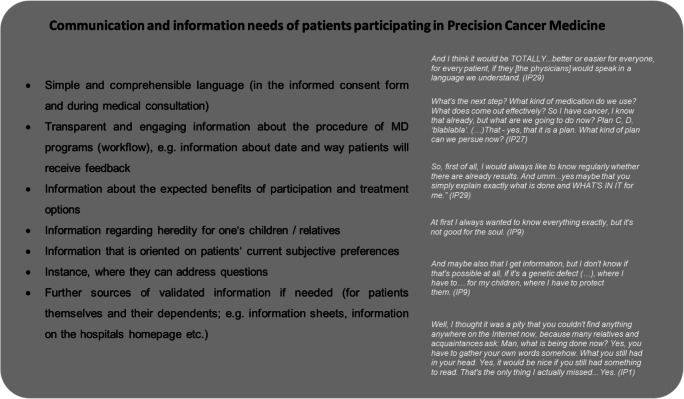
Summary of information needs patients explicitly stated throughout the interview

Over the course of the disease, for some patients, their need for information changed in general: While some mentioned that after some time they encouraged themselves to ask more questions about subjective implications of medical information, others described to have learned not to seek further information, because they were concerned that this information would cause them anxiety. Patients described that this change in information needs also to be applied to their information seeking regarding PCM. For example, patients mentioned that they would not go too much into detail, since they already had so many “turnarounds” (M110) that they were “not thinking about anything in advance” (M110) anymore and therefore experienced MD less agitatedly. Figure 2 presents a summary of information needs patients explicitly stated throughout the interview.

#### Uncertainties regarding personal benefits

Although in a lot of cases expectations were very high [12], many patients indicated their uncertainty about their actual chance to personally benefit by participating in the program. Some patients were not certain whether they would (merely) contribute to research or if and to what extent they could expect a personal benefit.

Well, I have a basic skepticism to the extent that I say, medically it’s all definitely meaningful and valuable, but whether something really ensues in the…for my specific case... let’s see. (IP10)

Yes, I’m missing it, uh, I’m actually missing...since I don’t know now, if there can still be an advantage for me at my age or maybe in ten years or whatever...in twenty years or something. Somehow...how shall I put it? Some purpose it will have in a way that medicine makes progresses. But whether it’s short term or long term, I don’t know. (…) Well, no I wasn’t actually told, but I guess it would help research and I doubt it’s gonna help ME. (IP17)

Many patients experienced this uncertainty as particularly burdensome while awaiting the results of the program.

So yes, and as I said, chemotherapy...is getting harder and harder to endure. So, at the moment the time factor is very...it is an important ...an issue since yesterday, right? (…) I can stand that [the waiting period] with chemotherapy, but...they [these months] will be exhausting. That is clear. I know that. (IP7)

This waiting time. Waiting, waiting, waiting. That is always the worst. That is ALWAYS the worst. (IP27)

Another patient described her perception while waiting for concrete results of PCM as “hanging in the balance”:

My son corresponded back and forth with Dr. A., and he was also always nice and positive, but he probably can’t say anything very concrete like that until this biopsy in [city 1]... we’re all pending there like everything is still hanging in the balance. (IP19)

#### Trust in the physician

Overall it became clear that throughout the course of the program, the trust in the physician played a central role for many patients participating in PCM. Regarding the reasons for participating in the program, one patient noted:

Well, that has a lot to do with trust, of course that is - for the patient it is the most important thing that you have trust in your doctor. There needs to be good chemistry, I can only speak for myself now, but I am sure that others feel the same…very important. (IP29)

Some mentioned that due to participating in the program, they finally had found a main contact person for their needs. Others pointed out that they had the feeling that the doctor “really cares about it and wants to do it [the PCM]” (IP9). Another patient stated that he liked the “feeling that the doctor is actually dealing with it [her case]” (IP4).

In many cases, it seemed that having the opportunity to participate in a novel research approach strongly influenced the trust in the treating oncologist:

So, I talked to Dr. B. too. And he also said to me: ‘Yes, we would do the same as the [hospital 1] doctors did. But there’s another study, there are also other things we can do.’ And I think that's what I missed [at hospital 1]. I’m somehow patient X there and here I have the feeling with Mr. B., that’s such a challenge, right? We want to defeat the cancer now or have it under control and that - I feel comfortable here somehow. (IP13)

In that regard, faith in research was evident in numerous interviews:

Well, I informed myself that there are really specialists there who are also investigating this in [City 1]. (…). And uh, they really are the absolute specialists and I hope they will find out what makes my therapy so ugh, so, as they say, so complicated, right? (IP14)

Furthermore, the trust in the physician seemed to “buffer” missing or incomprehensible information as well as uncertainty about any personal benefit.

(…) So far, I’ve understood everything I can imagine, how it should work, and the rest is uninteresting for me now, because I trust in the doctors, I have to say. (IP21)

It seemed that information exchange with the oncologist helped some patients deal with difficult feelings while awaiting the results of the PCM.

That always depends: If you don’t feel well, then you wish you could get information right now and immediately (laughs.). If things are good for you, then you don’t want to hear anything (laughs.) (…) because then simply this normality prevails again. So that’s not the case if you’re feeling bad, then you want the doctor to call you every hour and ask you how you're feeling. But if you are well, then not, then all that is gone. (IP29)

And Dr. C., I don’t know how many times she said that if I have questions, I can always ask her. So... I feel completely comfortable ...ugh…informed (*patient laughs*). (IP2)

## Discussion

Although patients felt well informed on average, many had to deal with a lack of PCM understanding. This has also been found in previous work [13]. A small number of patients had misinterpretations or questions regarding heredity and treatment options in case of a positive mutation result. In line with previous work on genomic counseling [24, 25], the findings of this study suggest that for patients participating in this complex program, it is enough to have a generic understanding of PCM, provided in a simple, comprehensible language. In addition, especially information concerning the overall process (e.g., time point of result disclosure) is important to provide orientation for the patients. According to patients’ need of transparent, continuous, and validated information and a defined medical contact, this study emphasizes the importance of providing appropriate infrastructure with validated information, e.g., by a PCM consultation hour. Moreover, misinterpretations and distress might be prevented if physicians anticipate “hot topics” — such as heredity — and address these with the patient in advance. In this regard, Goerling et al. [26] showed the bidirectional relationship between satisfaction with information and symptoms of anxiety.

Patients described the uncertainty while awaiting the results of the sequencing as burdensome, bearing in mind that most of the interviewed patients suffered from advanced disease and were exhausted from previous treatment. Overall, the existential threat seemed to be prominent apart from specific distress arising by participating in a PCM program.

In view of this, it seemed that patients’ trust in the physician buffered the psychological strain caused by the difficulties they experienced. This finding appears to be well substantiated by Shenolikar et al. [27] who found that a heightened vulnerability may increase trust in the physician. For some patients, the buffering effect of trust in their physicians was further underlined by a “faith in science”. In this regard, literature shows that beside a sense of caring and contextual factors (such as length of consultation), patients’ trust in their physician is mainly contingent by communicative aspects, competence, and honesty [28–32]. Furthermore, Atherton et al. [33] found that patients feel content with the information received when it is built on a caring relationship with their physician.

Although most patients expected a treatment benefit and thus obviously consented to participate (see also [12]), many seemed unaware of the small chances of an actual personal benefit. Here, it is paramount for oncologists to openly and empathetically discuss hopes and expectations as well as actual chances of a benefit from comprehensive genomic profiling with the patient. Furthermore, it seems crucial to strengthen physicians’ confidence in their genomic knowledge, as this was found to be not sufficient in other studies [34]. Additionally, in our view, it seems vital that patients get an idea about the complex interweaving of research and clinical practice. This should reduce misconceptions and enhance informed consent.

This study investigated patients’ perceptions on information regarding precision oncology programs. The manifold uncertainties arising from the evolving research approach of PCM are considerable for patient information and communication. Major challenges in PCM are to offer patients comprehensible information and guidance while supporting them to manage this vulnerable period of disease. This can be ensured by providing a continuous information transfer as well as transparent and standardized procedures. A communication based on a trusting relationship with the physician, which is built on competence and care, might offer a secure and reassuring counterpart against the uncertainties discussed. Moreover, information about the (for most cases) exploratory nature of PCM and the corresponding likelihood for a personal benefit needs to be openly and empathetically discussed, tailored to a patient’s current situation. Besides providing psycho-oncological support, we recommend the development of guidelines to help healthcare providers handle these challenges of patient communication in the growing field of PCM.

### Limitations

The findings’ generalizability is limited by the qualitative study design with the small sample size. Furthermore, the mean age of study participants was younger, and education level was higher compared with other studies [14], and thus, results may not be applicable for other patient populations. We recruited patients from different PCM approaches; however, the programs were rated as highly comparable by experts. The results gained were derived from qualitative interviews which explored patients’ subjective perception; no information material nor the informed consent talk has been analyzed to verify patients’ quotes.

## Electronic supplementary material

ESM 1 (DOCX 21 kb)

## Data Availability

The datasets generated during and/or analyzed during the current study are available from the corresponding author on reasonable request.
