# Supplementary material for: Information, communication, and cancer patients’ trust in the physician: what challenges do we have to face in an era of precision cancer medicine?
Source: Support Care Cancer. 2020 Sep 3;29(4):2171–8. doi: 10.1007/s00520-020-05692-7 (PMC7892511; doi:10.1007/s00520-020-05692-7)
Supplement: Supplementary file 1 — (DOCX 21 kb) [file 520_2020_5692_MOESM1_ESM.docx]

**Supplementary File 1: Interview Guide**

The interview guide was translated from German to English for publishing purpose.

1. How well-informed do you feel regarding this diagnostic / therapy program? Can you tell me something about that?
   1. You say that you feel well / poorly /… informed – so you have a specific / no / … picture of the processes around the diagnostic and therapy program? Are there perhaps open questions anyways? Where are you still lacking information?
   2. What steps are next?
   3. Where have you received your information from?
   4. Who is your person of contact [for this program]?
   5. All in all – how satisfied are you with your information?
2. Where would you locate your level of information about it on a scale from 0 = no information to 10 = very good information?
3. What personal expectations do you have regarding your “personalized diagnostic / therapy”? Can you tell me about this, too?
   1. You say that you have high / low expectations of this diagnostic / therapy. What exactly are you hoping for? Where are you skeptical?
   2. How did you come to participate in this program?
   3. What pros and cons are there for you?
   4. How are you doing in the current situation?
   5. What is the most helpful to you at the moment? What would you need in order to handle the current situation well?
